# Acute physical activity supports inhibitory control in primary school children: a randomised cross-over trial

**DOI:** 10.1038/s41598-026-44375-x

**Published:** 2026-03-30

**Authors:** Evelyn Watson, Paul W. Burgess, Isabel Metcalf, Mark Hamer, Flaminia Ronca

**Affiliations:** 1 Institute of Sport Exercise and Health, Faculty of Medical Sciences, University College London, London, UK; 2 Institute of Cognitive Neuroscience, Faculty of Brain Sciences, University College London, London, UK

**Keywords:** Cognitive neuroscience, Human behaviour

## Abstract

Studies have found inconsistent results regarding the acute effects of physical activity on inhibitory control in children. More naturalistic studies that have real-world validity and use objective measures of physical activity are essential. This study investigated the acute effects of a pre-existing school-based group physical activity session on inhibitory control. Fifty-five primary school children (9 ± 1 years old, 40% female) participated in a pre-existing school-based group physical activity session and a sedentary poster-making control condition, both lasting approximately 30 min, in a randomised, cross-over manner. Cognitive tasks were completed before and after both conditions, and measures of performance on these tasks were used as dependent variables in all analyses. Time engaged in moderate-to-vigorous physical activity during each condition was measured by accelerometry and used as a manipulation check. After physical activity there were faster reaction times (β = -176, 95%CI: -296.43, -59.51) and more commission errors (β = 1.06, 95%CI: 0.18, 1.95) in the simple reaction time task, but fewer commission errors (β = -4.64, 95%CI: -9.00 -0.28) in the inhibition task compared to the control condition. Using the same statistical approach, a supplementary analysis was run on a reduced sample of 32 (9 ± 1 years old, 44% female) whose adherence to conditions was confirmed by accelerometry. Results showed faster reaction times (β = -205, 95%CI: -384.58, -33.37) in the simple reaction time task and fewer commission errors (β = -6.10, 95%CI: -11.75, -0.45) in the inhibition task following physical activity. This suggests that an acute bout of physical activity facilitates inhibitory control in primary school children. Optimal effects are seen when participants achieved higher intensity physical activity during the intervention.

## Introduction

Inhibitory control is the ability to suppress prepotent responses in favour of more appropriate ones^1,2^. Inhibitory control develops rapidly during early childhood and has been shown to be important for school readiness and success^1,3^. In preadolescent children, inhibitory control has been positively linked to behaviour regulation^4^, on-task behaviour^5^ and academic outcomes^6^. Research has also associated traits of diminished inhibitory control, such as inattention and impulsivity, with poorer outcomes extending into adulthood, including lower socioeconomic status^7^, antisocial behaviour and substance abuse^8^. It is therefore important that the development of inhibitory control is supported throughout childhood.

There is general consensus that children should be physically active for optimal physical and cognitive health^9^. Reviews of the literature in children have found that while cognition generally benefits from acute moderate-intensity physical activity, effects are greater and more consistent for executive function, in particular inhibitory control^10^^,11^. This is corroborated by meta-analytic findings of a small to moderate effect of acute physical activity on inhibition in preadolescent children but no significant effect on other domains of executive function^12^. These results suggest a promising role for acute physical activity in supporting inhibitory control, although higher quality evidence is needed. Leveraging the benefits of physical activity is especially relevant in light of the global inactivity pandemic^13^. It is imperative that feasible and effective solutions to optimise the cognitive benefits of physical activity are found and implemented.

Time at school accounts for the majority of children’s daily sedentary time^14^ and is a promising environment to increase physical activity. A body of research has investigated school-based physical activity interventions. Results have been mixed regarding the acute effects of academically unrelated physical activity on inhibitory control in pre-adolescents. There has been evidence for improved inhibition following 20 min of cognitively engaging physical activity^15^. Other research has found only trends for improved inhibition after 15–20 min of simple aerobic physical activity^16^, or failed to find improved inhibition following 20 min of physical activity, whether simple aerobic or with a cognitive component^17,18^. The often limited resources available to schools for physical activity^19^ make it essential to understand where resources should be placed for interventions to have greatesimpact.

A potential reason for discrepancies in results is variation in the physical activity, samples investigated and cognitive tasks used, resulting in incomparable methodologies^20^. For example, the extent to which physical activity is cognitively engaging, which has received increasing interest as a determinant of impact on executive function, is a somewhat subjective and qualitative characteristic of physical activity, difficult to measure and compare between studies^21^. More objective characteristics such as activity duration^22^, intensity^23^, fitness^15^ and body composition^24^^25^, have also been shown to impact the association between acute physical activity and inhibition. Limited research has investigated the role of intensity on the impact of acute physical activity on inhibition in preadolescent children. It has been recommended that future studies use accelerometry to assess physical activity dose at the individual level^26^.

Understanding the impact of acute physical activity on inhibitory control will inform how real-world interventions may be optimised to benefit children. There is a lack of naturalistic studies with real-world validity and using objective measures of physical activity in the existing literature. The aim of the current study was therefore to compare the acute effects of a pre-existing school-based group physical activity session to a sedentary, cognitively engaging group activity on inhibitory control in primary school pupils, measured objectively via performance on cognitive tasks. It was hypothesized that inhibitory control would improve following the physical activity session compared to the control condition. The physical activity session of interest is an extracurricular multisport session provided as part of a school-based physical activity program run by a charity. Sports coaches are placed in schools in disadvantaged communities to support the development of young people via physical activity opportunities. In primary schools this consists of structured physical activity before, during and after the school day, independent from the academic curriculum. The emphasis is placed on personal development and instilling in young people a lifelong enjoyment of being active.

## Methods

### Participants

A total of 71 typically developing children aged 8 to 11 took part in the study. Of these, 55 (9 ± 1 years old, 40% female) completed both conditions, consisting of a physical activity session and a sedentary control. Participants were recruited from four primary schools in London, United Kingdom, following the consent of headteachers. Informed consent was obtained from parents or legal guardians of participating pupils. An a priori sample size calculation for a repeated measures, within factors ANOVA was run using G*Power 3.1.9.7^27^. Based on the effect size found in a similar study (η^2^*p* = 0.04^15^, a sample of 35 participants was required to reach a power of 0.80 at an alpha level of 0.05, with one group, four measurements and correlation among repeated measurements set to the default 0.5. More participants than required were recruited to account for dropouts, missing data and exclusions.

### Procedure

Data collection took place in primary schools between February 2023 and February 2024. A within-subjects design was used whereby participants completed both conditions, on separate days, in a cross-over, randomised manner. Researchers visited the schools during extracurricular clubs lasting approximately 30 min and each child completed both conditions at the same time of day. Cognitive tasks took approximately 10 min and were completed immediately before and after both conditions. All participating pupils completed them at the same time, using school computers or laptops in a quiet classroom. Researchers fitted pupils with an accelerometer before they took part in each condition. After completion of the post-condition cognitive tasks, participant height and waist circumference were measured. Ethical approval was granted by the University College London Research Ethics Committee (722/002) in line with the declaration of Helsinki.

### Experimental conditions

#### Physical activity

The physical activity condition was a pre-existing extracurricular session run by a coach as part of a wider physical activity program. Session attendance varied from 5 to 20 pupils, including those not participating in the current study. The sessions took place either just before or after the school day, on the same day each week throughout each school term and either in a sports hall or outdoors depending on the weather. Each session focussed on a specific sport or game for the entire term. The sport played were football (soccer), basketball and dodgeball, chosen based on what was popular with pupils. Emphasis was placed on enjoyment and participation in physical activity over high performance.

#### Sedentary activity

The sedentary activity involved participants creating posters about their favourite sports player. This activity was completed in groups of between 5 and 10 pupils (dependant on session attendance) in the same classroom where cognitive tasks were completed. Researchers introduced the activity by asking pupils who their favourite sports players were, illustrating the diversity of opinions. Pupils were told to make a poster persuading their classmates that their favourite sport player was the best. Before beginning, researchers showed participants an example poster. Throughout the activity, researchers made sure pupils remained on-task and asked them about their sports player and poster.

### Outcome measures

#### Cognitive tasks

**Fig. 1:**
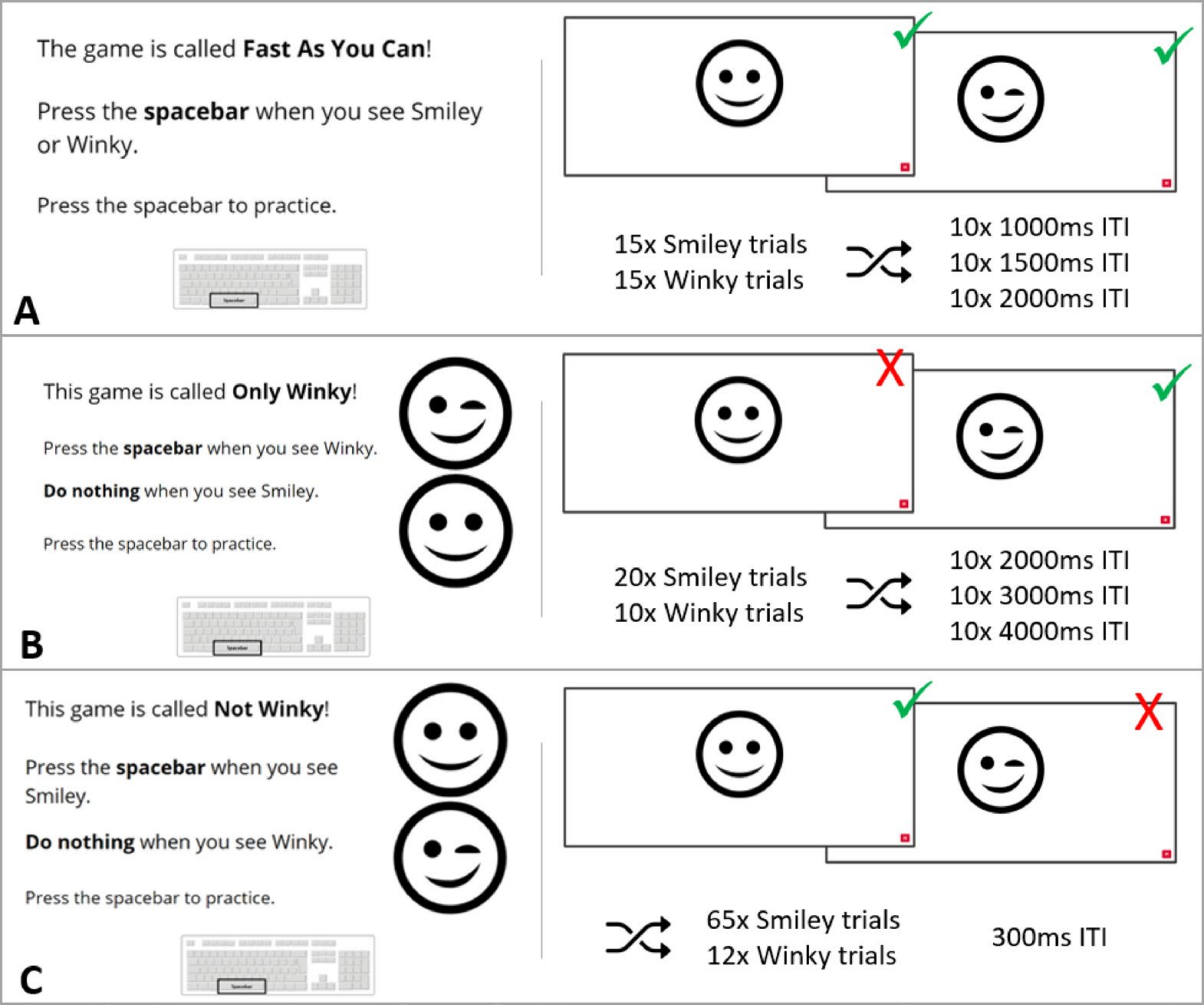
Stimuli and instructions for the (**A**) simple reaction time, (**B**) sustained attention and (**C**) inhibition tasks. Adapted from Watson et al.^30^.

A battery of computerized cognitive tasks (Fig. 1) was used to measure inhibitory control. This battery was created and hosted using the Gorilla Experiment Builder^28^ and has been previously validated with a high test-retest validity, demonstrating sensitivity to age^29^, including with this age group^30^. The battery consisted of three tasks: a simple reaction time task, a sustained attention task and an inhibition task. As per the use of these tasks in prior work^29,^^30^, they were administered in the same order for all participants, designed to maximise their construct validities. This order was: simple reaction time task, sustained attention task and inhibition task. Stimuli across all tasks were smiley and winky faces of consistent size, presented in a pre-randomized order in the centre of the screen. The simple reaction time task required participants to press the spacebar on their keyboard as fast as possible without making mistakes when either a smiley or winky face appeared. There were eight practice trials followed by thirty task trials, split evenly between smiley and winky faces and inter-trial intervals of 1000ms, 1500ms and 2000ms. The sustained attention and inhibition tasks were Go/NoGo tasks. In the sustained attention task, participants were instructed to press the spacebar as fast as possible without making mistakes when a winky face appeared, but to do nothing when a smiley face appeared. The task consisted of eight practice trials followed by thirty task trials. There were ten ‘Go’ winky trials and twenty ‘Stay’ smiley trials with inter-trial intervals of 2000ms, 3000ms and 4000ms. The inhibition task contrasted in that participants were instructed to respond to smiley trials but not winky faces. The task consisted of 74 trials (62 smiley and 12 winky) preceded by eight practice trials, with all inter-trial intervals of 300ms.

For all tasks, mean reaction time speed was calculated by removing responses faster than 125ms, following standard practice^31^, and averaging across all correct trials. Commission errors were calculated as the number of trials where a response was incorrectly given during the inter-trial intervals, including responses to stimuli faster than 125ms. Stay and NoGo errors were calculated for the sustained attention and inhibition tasks respectively as the number of Stay or NoGo trials where a response was incorrectly given, excluding responses faster than 125ms^24,32^.

#### Accelerometry

ActiGraph wGT3X-BT accelerometers were worn on the waist^33^, under the non-dominant armpit with the charging port facing upwards. The sampling rate was set to 100 Hz and data was downloaded with epoch set to one second and number of axes to three. Accelerometry was chosen over heart rate as an objective measure of physical activity as parents of participants would not have consented to the more intrusive requirements of a heart rate chest strap compared to an accelerometer worn on top of clothes. In addition, the use of accelerometry within translational physical activity research with children is supported by reviews in the field^21^.

### Data analysis

Data manipulation and analysis were carried out using R Studio (4.2.1). Participant data was matched across conditions using pseudonymous identification codes. Accelerometry data was analysed using the ActiLife software v6.12.1. Freedson 1998 cut points^34^ were used to determine percentage of time spent in moderate to vigorous physical activity (MVPA). Wear-time validation used the Troiano 2007 algorithm^35^. Internal consistency of the cognitive tasks was assessed using Guttman bounds from split-half reliability testing (λ3 and λ4). Data were tested for normality using a Shapiro–Wilk test^36^. A main analysis of the acute effects of physical activity on cognition was carried out using ANCOVAs. Condition was the independent variable and performance on cognitive tasks at the post-condition timepoint was the dependent variable. To control for baseline imbalances, performance at the pre-condition time point was a covariate. Analyses were also run including time of day as a covariate, however this did not impact results. Where dependent variables were non-normally distributed, results were verified by repeating the ANCOVAs following Box-Cox and rank transformation of continuous and discrete dependent variables respectively. Reported results are from analysis of non-transformed data. A supplementary analysis was run, using the same statistical approach, on a reduced sample size where adherence to both conditions was confirmed by accelerometer data. In other words, the main analysis simply required participants to have complete cognitive task data, consisting of pre- and post- timepoints for both conditions. Inclusion in the supplementary analysis required participants to have complete cognitive task data as well as accelerometer data for both conditions which confirmed that their behaviour reflected how these were designed (i.e. sedentary versus physically active). The alpha level for all tests was 0.05.

## Results

### Demographics

Two participants were removed prior to analysis due to high numbers of commission errors, suggesting a lack of understanding or desire to complete the tasks correctly. The main analysis was therefore completed on 53 participants (9 ± 1 years old, 42% female, waist-to-height ratio 0.47 ± 0.06). A total of 52 children consented for accelerometry data to be captured. Of these, 43 had accelerometry data collected during both conditions and were included in the manipulation check. For the supplementary analysis, participants were excluded if they did not have accelerometry data for both conditions or if their time in MVPA was an outlier for either condition, considered as lower than 29% for the physical activity condition and greater than 16% for the control condition, based on visual inspection of histograms. Of the participants included in the main analysis, two were excluded as more than 20% of the control condition was spent in MVPA, 18 were excluded due to missing accelerometry data for one of the conditions and one was excluded due to lack of consent for accelerometry to be captured. Therefore, a sample of 32 participants (9 ± 1 years old, 44% female, waist-to-height ratio 0.47 ± 0.06) was analysed for the supplementary analysis.

### Reliability testing

Internal consistency was good on all tasks. λ3 was 0.87, 0.71 and 0.93 and λ4 was 0.98, 0.84 and 0.97 for the simple reaction time, sustained attention and inhibition tasks respectively.

### Manipulation check

**Fig. 2:**
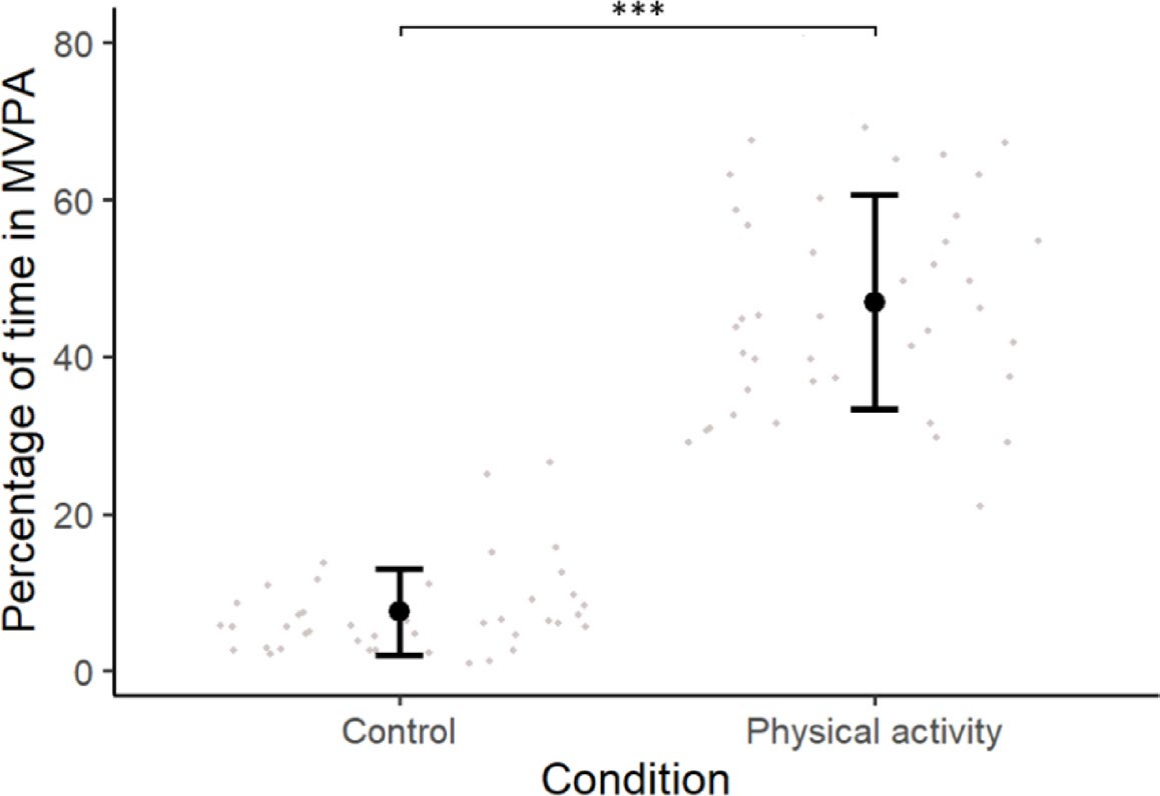
Mean percentage of time in moderate to vigorous physical activity (MVPA; ± sd) in both study conditions (*n* = 43, *** *p* <.001).

The mean session length (23.9 ± 6.3 min) to be significantly greater (*p* <.001) for the physical activity (27.2 ± 5.9 min) compared to the sedentary control (20.7 ± 4.9 min). Within-subjects comparison between conditions showed a significantly greater (*p* <.001, effect size *r* =.87; Fig. 2) percentage of time spent in MVPA during the physical activity condition (47.2 ± 13.1%) compared to the sedentary condition (7.2 ± 5.5%).

### Main analysis

**Fig. 3:**
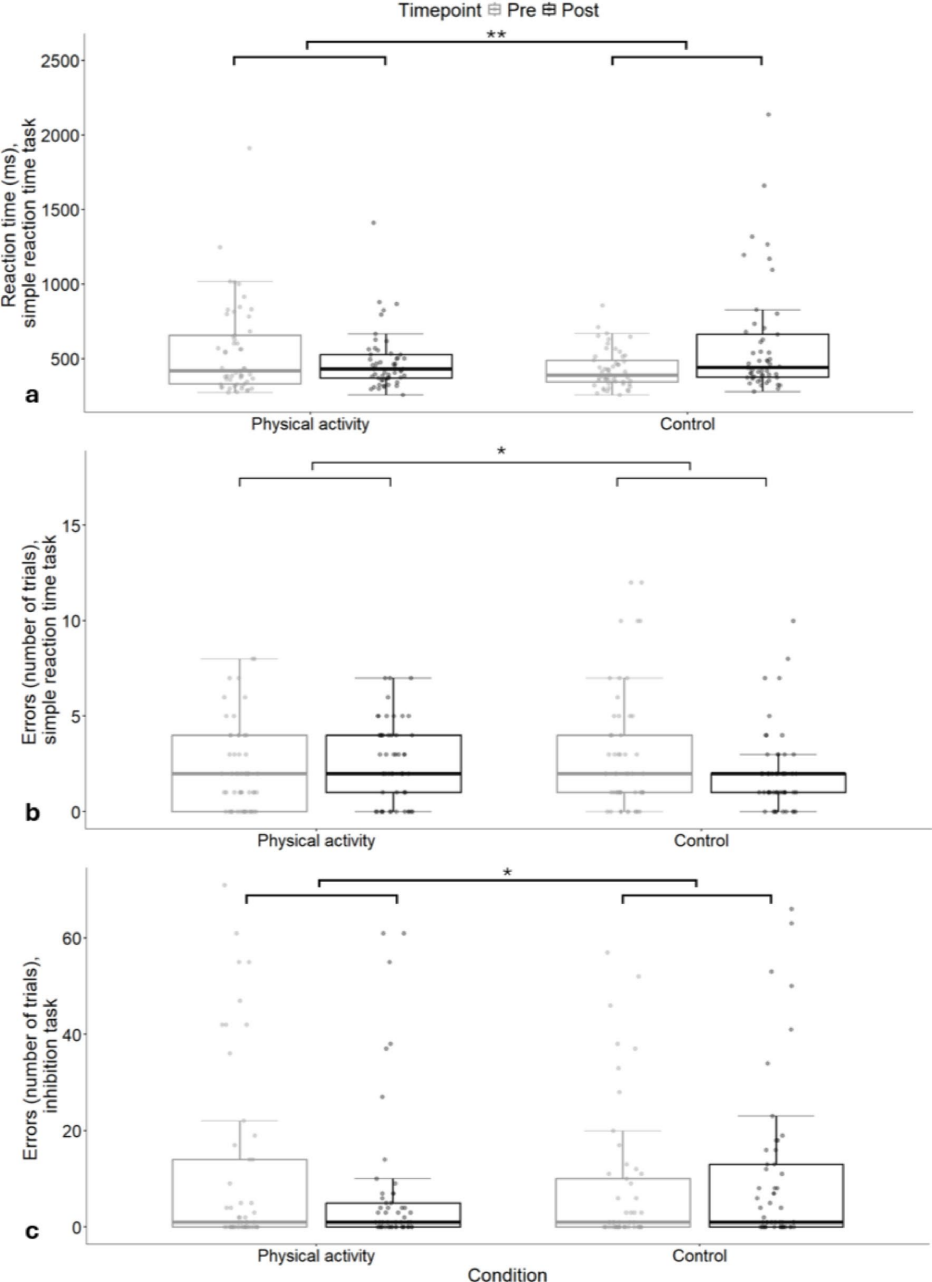
Mean reaction times (**a**) and commission errors (**b**) in the simple reaction time task and error trials in the inhibition task (**c**), by condition and timepoint, as per the main analysis (*n* = 53, ** *p* <.01, * *p* <.05).

In the simple reaction time task (Fig. 3a and b), results showed a significant difference between conditions for mean reaction time post-condition after adjusting for pre-condition reaction times (β = −176, 95%CI: −296.43, −59.51, *p* =.005). Reaction times were slower following the control condition (640 ± 48 ms) compared to the physical activity condition (464 ± 48 ms). There was also a significant difference between conditions for commission errors made post-condition, after adjusting for pre-condition commission errors (β = 1.06, 95%CI: 0.18, 1.95, *p* =.02). Significantly more commission errors were made following the physical activity condition (2.99 ± 0.33) compared to the control (1.93 ± 0.33).

In the inhibition task (Fig. 3c), there was a significant difference between conditions for commission errors made post-condition, after adjusting for pre-condition commission errors (β = −4.64, 95%CI: −9.00, −0.28, *p* =.04). Significantly fewer commission errors were made following the physical activity condition (6.37 ± 1.58) compared to the control condition (11.01 ± 1.58). No significant differences were found for reaction time or NoGo errors. No significant differences between conditions were found for any measures of performance in the sustained attention task.

### Supplementary analysis

**Fig. 4:**
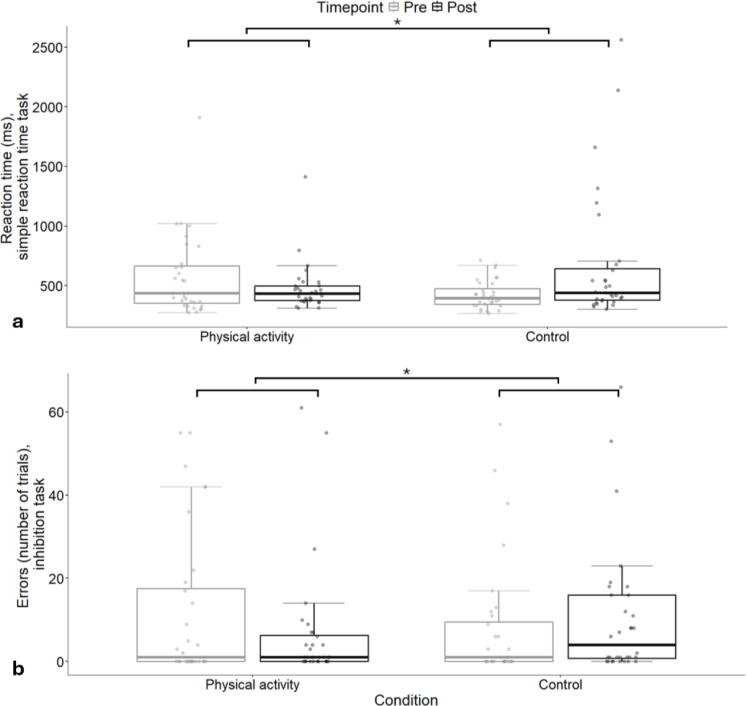
Mean reaction times in the simple reaction time task (**a**) and error trials in the inhibition task (**b**), by condition and timepoint, as per the supplementary analysis (*n* = 32, * *p* <.05).

In the simple reaction time task (Fig. 4a), results showed a significant difference between conditions for mean reaction time post-condition after adjusting for pre-condition reaction time (β = −205, 95%CI: −384.58, −33.37, *p* =.03). Reaction times were significantly slower following the control condition (677 ± 74.2 ms) compared to the physical activity condition (472 ± 74.2 ms). No significant differences were found for commission errors in the simple reaction time task. In the inhibition task (Fig. 4b), there was a significant difference between conditions for commission errors made post-condition, after adjusting for pre-condition commission errors (β = −6.10, 95%CI: −11.75, −0.45, *p* =.04). Fewer commission errors were made following the physical activity condition (5.65 ± 2.05) compared to the control condition (11.75 ± 2.05). No significant differences were found for reaction time or NoGo errors. No significant differences between conditions were found for any measures of performance in the sustained attention task.

## Discussion

This study compared the acute effects of physical activity and a sedentary activity on inhibitory control in primary school children, as measured by performance on cognitive tasks. Uniquely, this is one of the few studies to have investigated a pre-existing school-based physical activity program, highly naturalistic and with known real-world feasibility. A main analysis showed fewer commission errors in the inhibition task following physical activity compared to the control condition, as well as greater impulsivity in the simple reaction time task, as determined by faster reaction times with more commission errors. A supplementary analysis on a reduced sample whose adherence to both conditions was confirmed via accelerometry showed faster reaction times in the simple reaction time task and fewer commission errors in the inhibition task following the physical activity condition. This suggests that acute physical activity in the naturalistic setting benefits inhibitory control in primary school children, assuming they engage in the physical activity to accrue time in MVPA.

In the simple reaction time task, the supplementary analysis showed faster reaction times following the physical activity condition compared to the control condition. In existing literature, studies by various groups have also found an improvement in reaction time following physical activity^24,32,37,38^. However, there is a dearth of studies investigating acute effects of specifically school-based physical activity on simple reaction time^21^. The current study therefore contributes ecologically valid evidence. Indeed, prior studies differ from the current naturalistic, group physical activity session by being lab-controlled and using exergaming and cycling, both individual activities, with the former lasting only 10 min and lacking control condition. In the current study there may also have been greater variation in the delay between end of physical activity and post-condition testing. This again makes it more ecologically valid when considering the real-world benefits of inhibitory control following physical activity, for example classroom behaviour and school attainment^5,6^. However, differences between the main and supplementary analyses must be interpreted with caution to sample size differences. It may be that acute physical activity did indeed increase impulsivity in the simple reaction time task but improve inhibitory control as measured by the inhibition task, suggesting that physical activity differentially affects cognitive performance.

Results showed acute physical activity to benefit inhibitory control in primary school children. This is largely in line with wider literature, mostly from laboratory-controlled studies^11^, but contradicts certain more methodologically similar studies. For example, a naturalistic study by Jäger and colleagues found non-significant effects of physical activity, either cognitively engaging or non-engaging, on inhibitory control^18^. The wide range in participant heart rate during physical activity has been discussed as an explanation for the lack of effect^18^. As in the current study, and a challenge of all naturalistic studies, not all participants engaged with the physical activity to the same extent or in the same manner. It would have been interesting if Jäger and colleagues had also carried out their analysis on a sub-sample who engaged adequately with the physical activity. Other studies which found no benefits to inhibitory control following acute physical activity have also discussed low intensity of physical activity as a potential limitation^17^. As has previously been recommended^12^, future research should compare physical activity conditions of different intensities to determine the optimal physical activity intensity to support inhibitory control in pre-adolescent children. This should occur alongside behaviour change research to ensure that all children engage with and benefit from this physical activity.

Research has shown the importance of physical activity characteristics for its impact on cognition^39^. The physical activity sessions in the current study were group-based multisport sessions with an emphasis on enjoyment and being active, not performance. Team sports included in this type of physical activity session are social and involve a number of cognitive processes. It is likely that the effect on inhibitory control is not purely a result of the physiological effects of running around. For example, research has suggested that the extent to which physical activity is cognitively demanding plays a role in its impact on cognition^40^. However, the control condition was designed to also be cognitively engaging, allowing the current study to investigate the acute effect of a physical activity program specifically. The extent to which an environment is sociable and the manner in which participants interact during physical activity may also be critical to the acute impact on executive function. A systematic review of randomise controlled trials concluded that the biggest effect sizes of physical activity on cognition were in studies using team games, although these were interventions^41^. It remains unclear what the hierarchy of importance may be between these and other characteristics of physical activity, and whether this is dependent on cognitive domain or individual differences between participants.

The main shortcoming of the study was the reduced sample size in the supplementary analysis, largely due to missing accelerometry data. Owing to the naturalistic design there was greater variability in individual physical activity intensity (time in MVPA) than might have been expected in a controlled laboratory study. In addition, a side effect of investigating physical activity within a pre-existing program, participants invited to consent were already signed up to participate in the sessions. Although partially mitigated by the parental incentive of free childcare, the sample may have been biased towards children who enjoy physical activity. There may therefore have been differences in intrinsic motivating factors between conditions, influencing engagement with the sessions and desire to carefully complete cognitive tasks. Future studies might assess enjoyment of each condition to include as covariate, or match enjoyment between conditions^15,42^. Other confounders which vary day-to-day, for example mood, might also be controlled for^34^. Similarly, the fact that different sessions across the study focussed on different sports may have influenced results, for example due to varying participant familiarity with the game or complexity of motor skills. Furthermore, the significant difference in duration between conditions may have influenced results and future studies should put in place measures to avoid this. Considering possible differences in enjoyment between conditions, blinding the condition until after pre-condition cognitive task completion may avoid child-driven delays for example in collecting belongings and moving to the room where the cognitive tasks and control condition took place.

## Conclusions

The current study found improved inhibitory control following engagement in acute naturalistic physical activity compared to a sedentary control condition in primary school children. The strength of this research’s ecological validity and objective measures of physical activity and inhibitory control should be highlighted. This has important implications for the implementation and monitoring of real-world physical activity interventions. In particular, this suggests that a model focused on structured, non-academic physical activity provided by specialist coaches in deprived primary schools can improve objectively measured inhibition performance. However, it may not be sufficient to provide children with opportunities for physical activity, but crucial that children engage with them. What this engagement looks like, how to drive it and monitor it are important questions requiring future investigation.

## Data Availability

The data and analytic code necessary to reproduce the analyses presented here are not publicly accessible but can be made available by the authors upon request, by contacting [evelyn.watson.22@ucl.ac.uk](mailto: evelyn.watson.22@ucl.ac.uk).
